# High Prevalence of Nephrocalcinosis in Hypophosphatasia Patients with the *ALPL* c.1559del Gene Variant

**DOI:** 10.31662/jmaj.2024-0138

**Published:** 2024-12-20

**Authors:** Hisashi Kawashima, Atsuko Sasame, Yoko Ogaki, Takayuki Nakayama

**Affiliations:** 1 Department of Pediatrics, Kohseichuo General Hospital, Tokyo, Japan; 2 Department of Internal Medicine, Kohseichuo General Hospital, Tokyo, Japan; 3 Department of Orthopedics, Kohseichuo General Hospital, Tokyo, Japan; 4 Department of Pediatrics and Adolescent Medicine, Tokyo Medical University, Tokyo, Japan

**Keywords:** Hypophosphatemia, Ionized Ca, Bone mineral density, Urine Ca/Cr, Nephrolithiasis

## Abstract

**Introduction:**

Hypophosphatasia has been reported to develop nephrocalcinosis, renal stone, and chronic kidney failure. We investigated their renal impairments in the adults with hypophosphatasia to know the phenotype-genotype correlation

**Methods:**

We subjected 11 patients with hypophosphatasia who were diagnosed by chance in the routine medical health checkup. Most cases had past history of fracture. Bone mineral density showed low or lower normal limit.

**Results:**

Four of six patients also had high levels of ionized Ca. In subjected six cases, four showed high urinary Ca excretion. Nephrocalcinosis is found in five cases even if the symptoms of hypophosphatasia are mild. Four out of five patients with a mutation of c.1559del in *ALPL* had nephrocalcinosis and/or kidney stones. One patient already developed hydronephrosis. One of six patients with other mutations showed nephrocalcinosis.

**Conclusions:**

The phenotype-genotype correlation between renal impairment and c.1559del of *ALPL* gene was suggested.

## Introduction

Hypophosphatasia (HPP) is a rare metabolic disease in which an enzyme called alkaline phosphatase (ALP) becomes inactive due to variants of *ALPL* ^(1), (2)^. ALP is widely distributed throughout the body, such as the liver, kidneys, osteoblasts, and small intestine, and mostly localized on the cell membrane that is released into the serum. Therefore, serum ALP levels are low in patients with hypophosphatasia (HPP). HPP is divided into six types, and the severe perinatal form occurs in about 1 in 150,000 births. The rate of gene carriers is suspected approximately 1 in 600 in Japan ^(3)^. Symptoms of carriers or patients with heterozygous remain unclear. Patients with HPP may show normal ALP levels when they have a fracture, and children generally show higher serum ALP levels than adults because they are growing. Therefore, mild cases of HPP are often overlooked for a long time until the patients reach adulthood. As adult HPP is a mild form that presents with unspecific signs, such as osteopenia, osteomalacia, muscle involvement, and headache, patients are misdiagnosed as having osteoporosis on routine examinations ^(1)^. The major symptoms of adult HPP are the presence of musculoskeletal pain (100% of patients) and muscular weakness (83.3% of patients) ^(4)^. Moreover, HPP has been reported to develop nephrocalcinosis, renal stone, and chronic kidney failure ^(5), (6)^. We identified 11 patients who were diagnosed by chance as having HPP at their usual medical health checkup due to hypophosphatemia. Most patients had been experiencing frequent fractures. At present, knowledge about the phenotype-genotype correlation of HPP is restricted to its bone manifestations ^(7), (8)^. The association between *ALPL* genotypes and renal symptoms remain unclear, particularly in Japan, although urinary Ca excretion is suspected to increase depending on the genomic variant ^(9)^. We investigated the renal impairments of adults with HPP, to clarify the phenotype-genotype correlation.

## Materials and Methods

### Criteria of screening for HPP

Between September 2022 and August 2023, 13,550 people (8,607 men and 4,943 women) who visited our health checkup center were screened for HPP. The eligibility criteria of age of the subjects were 20-80 years old. A total of 79 people (49 men and 30 women) were identified as having an ALP level of less than 33 U/L for men and less than 29 U/L for women (ALP general standard: 38-113 U/L for both men and women), considering gender differences. In the clinical setting, adult HPP is screened as follows: (1) low levels of ALP; (2) those who have a history of fractures, those who have constant fatigue; and those who have joint or muscle pain of unknown cause; and (3) as it is a hereditary disease, even if the person himself/herself is asymptomatic, the presence of the following symptoms in family members: (a) early loss of deciduous teeth (lost at the age of 1-4 years), (b) short stature, (c) gait disorder, or (d) easy fractures. A written recommendation for a detailed medical examination was provided to these individuals. A total of 14 people (7 men and 7 women) subsequently consulted the genetic outpatient clinic. We found that 11 out of the 14 patients had genetic variants and the characteristics that were compatible with HPP (1 is carrier) ^(9), (10)^. We also measured serum Ca, ionized Ca (iCa), and urinary calcium/creatinine (UCa/Cr) in those patients who were diagnosed as having HPP.

Gene variant analyses were performed in both the exons and introns (inside ten nucleotides) of *ALPL* by target next-generation sequencing using hybridization capture. The obtained results were compared with human genome references (GRCh38/hg3) for low-frequency variants, deletions, and insertions, through a commercial company (Kazusa Institute, Chiba, Japan).

## Results

### Their clinical symptoms and laboratory findings

Table 1 shows that their symptoms associated with HPP were mild or absent. The numbers of fractures experienced were none to ten times without any problem with healing since childhood. Five patients showed low levels of serum 25-hydroxy vitamin D. Genomic studies of *ALPL* showed c.1559del (p.Leu520ArgfsTer86) in five cases, c.529G>A (p.Ala177Thr) in two cases, and c.529G>A/c.1559del, c.613G>A (p.Ala205Thr), c.979T>C (p.Phe327Leu), and c.1171dup (p.Arg391ProfsTer14) in each having one case. Of the 14 subjects, 3 showed no pathogenic variants in *ALPL*. Two subjects were diagnosed as having secondary HPP due to their low blood Zn levels.

**Table 1.** Characteristics of the Patients and *ALPL* Gene Analysis.

No.	Age	Sex	Complaint	Fracture	ALP (normal range: 38-113) (U/L)	Ca (normal range: 8.8-10.1) (mg/dL)	iCa (normal range: 2.41-2.72) (mEq/L)	Urine Ca/creatinine (normal range: <0.1) (g/g・Cre)	25 OH-vitamin D (normal range: >20) (ng/L)	Abdominal echo or CT	BMI lumbar spine/femur (%)	Genomic study (all heterozygous)	Diagnosis
1	44	F	None	0	**30**	9.4	n.d.	n.d.	**12.4**	Normal image	96/91	**c.1559del**	Carrier
2	62	M	False aneurysm	1	**23**	9.7	n.d.	n.d.	n.d.	Renal cyst (l)	129/90	c.613G>A	Adult type
3	49	M	Muscle pain, migraine arthralgia (knee joints)	0	**19**	9.9	**2.92**	**2.9/27.16**	n.d.	Normal image	110/87	c.529G>A **c.1559del**	Adult type
4	37	M	Migraine arthralgia (hip joint)	1	**29**	9.5	n.d.	n.d.	n.d.	Normal image	118/108	c.529G>A	Adult type
5	61	F	Kidney calcification	0	**27**	9.5	**2.76**	**16.9/80.91**	**19.1**	Renal cyst **calcification**	92/76	**c.1559del**	Adult type
6	53	M	None	10	**28**	9.3	**2.84**	n.d.	n.d.	Renal cyst (r and l)	87/73	c.1171dup	Pediatric type
7	30	F	Muscle pain migraine arthralgia	1	**33**	9.6	2.69	n.d.	**12.6**	n.d.	85/90	c.529G>A	Adult type
8	53	M	None	3	**29**	9.5	2.61	7.2/110.83	20.9	**Calcification**	77/79	c.979T>C	Adult type
9	59	M	None	2	**19**	9.6	2.72	**4.5/25.46**	**19.8**	Renal cyst **kidney stones**	81/85	**c.1559del**	Adult type
10	56	M	Hyperlipidemia, heart valve disease, enthesopathy	0	**20**	9.3	2.63	**19/106.9**	28.9	**Calcification**, **kidney stones**, hydronephrosis	140/105	**c.1559del**	Adult type
11	54	F	Hypoplasia of tooth enamel	2	**36**	9.8	**2.79**	2.4/29.09	48.6	Calcification	109/97	**c.1559del**	Adult type
Reference ^(26)^	0	M	Short stature, respiratory failure, muscle weakness, no calcification of skull	0	**12**	10.4				**Kidney stone**	Not done	**c.1559del**/ c.1276G>A	Perinatal type

n.d., not determined; *reference values of perinatal HPP patients in our institute; bold, abnormal data

### Renal involvements in patients

**Figure 1.**
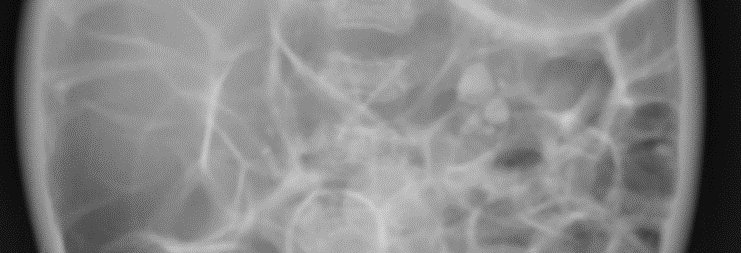
Abdominal X-ray: X-ray represents renal stone in the left kidney.

**Figure 2.**
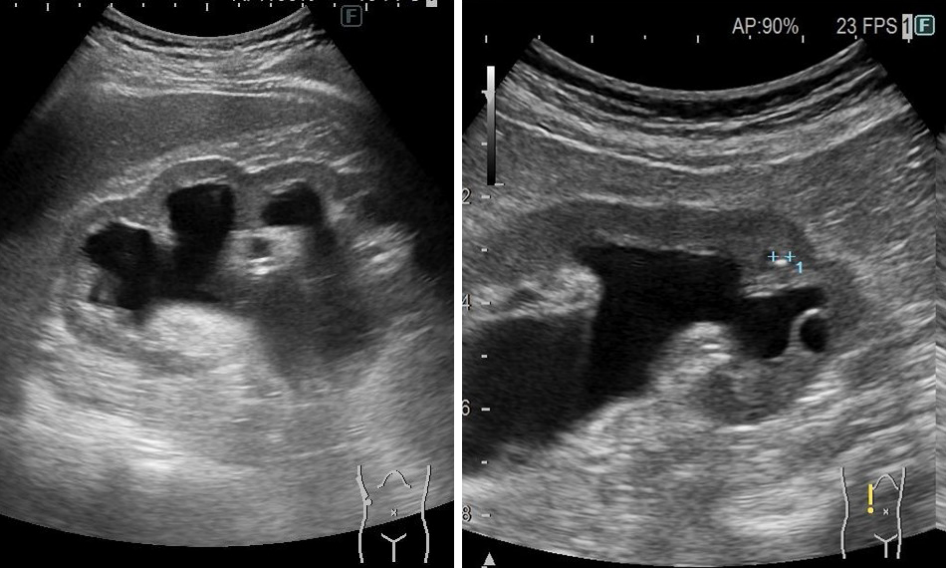
Ultrasound findings of the right kidney: nephrocalcinosis and hydronephrosis in the patient who has heterogynous variant of c.1559del.

Four out of six patients also had high levels of iCa. Four out of five patients with a heterozygous variant of c.1559del in *ALPL* had nephrocalcinosis and kidney stones (Table 1 and Figure 1). One patient also developed hydronephrosis (Figure 2). Of the 13,550 individuals in the entire target group, 3,028 (22.2%) had renal calcification or kidney stones. The Fisher test showed a statistically significant difference of 0.00273 (*p* < 0.05) between patients with *ALPL* c.1559del and all other individuals. By contrast, only one showed nephrocalcinosis in five patients with other pathogenic variants. Two patients with heterocompound variants with c.1559del showed nephrocalcinosis in a perinatal patient.

## Discussion

HPP is generally characterized by severe skeletal symptoms, such as mineralization defects, insufficiency fractures, and delayed healing of fractures. Some patients are thought to develop chronic renal failure secondary to hypercalcemia/hypercalciuria, the ingestion of a toxic agent (NSAIDs, bisphosphonate, etc.), and/or hyperphosphatemia (increases renal tubular phosphorus reabsorption) ^(11)^. In the most severe perinatal type, renal calcification was reported to occur within 6 months after birth similar as our perinatal case. Of the 52 cases in Japan, only 3 with renal calcification were reported to be only perinatally fatal type ^(12)^. This study investigated that renal calcification occurs frequently in adults having c.1559del including carriers (cases with normal bone density). Urinary Ca/Cr is elevated in most subjected cases. Association renal impairment with c.1559del of *ALPL* in adults with hypophosphatasia is overlooked for a long time. In our study, one perinatal case with renal stone also has c.1559del. Taketani et al. examined the clinical and genetic in Japanese 31 pediatric patients with HPP. The first and second most frequent variant genes were c.1559delT and c.T979C (p.F327L) in the *ALPL*, respectively. They suggested that different clinical features were associated with the same genotype in the non-lethal type ^(12)^. HPP’s remarkably broad-ranging expressivity is largely explained by autosomal recessive versus autosomal dominant transmission from among several hundred, usually missense, *ALP* variants. Since two adult patients had relatives with kidney diseases in their family history, autosomal dominant transmission was suspected (data not shown). Whyte et al. assessed their 25-year experience with 173 pediatric HPP patients. Most patients represented autosomal dominant inheritance of HPP. Mutant allele dosage generally indicated the disorder’s severity. Gender discordance was found for severe childhood HPP, 42 boys versus 16 girls, perhaps reflecting parental concern on stature and strength ^(13)^. In our study, as most cases including perinatal type who had renal calcification were male, there might be also gender difference on renal calcification.

Each patient had a different genotype. The high genetic variability of *ALPL* results in high clinical heterogeneity ^(10), (14)^. Recent genomic analyses have identified a mild form of HPP that appears to be relatively common. The hallmark of this mild form is a persistently low serum ALP. The correlation between their phenotype and specific variants that we found in this study might be due to different activity of kidney Tissue ALP. The activity of ALP by c.1559del (p.Leu520Argfs) is reported to decrease as 5.6 ^(15)^ or 28.8% ^(16)^. Since c.1559del is the most common variant in HPP in Japan. It was reported that conventional abdominal plain films fail to detect nephrocalcinosis in HPP ^(17), (18)^. Therefore, patients who have those variants is recommended to be examined through ultrasound every 3-5 years even if there are no or mild symptoms ^(19)^.

The pathophysiology of nephrocalcinosis in HPP remains unclear ^(10)^. In our study, most subjected patients showed high levels of serum iCa. Therefore, hypercalcemia is suspected to be pivotal due to nephrocalcinosis. In autopsy cases, the kidneys showed calcium deposits in the cortical interstitium and calcified foci were scattered in the cortico-module zone. Moreover, ultrastructural observation showed that calcium deposits were seen only in the epithelial cells of the distal tubules and Henle’s loop ^(20)^.

Enzyme replacement therapy using asfotase alfa is shown as a promising treatment for HPP ^(21), (22), (23)^. During clinical trials, there was no progression of nephrocalcinosis in infantile HPP, and the status improved in some patients ^(24)^. Indication of enzyme replacement therapy using asfotase alfa involves radiological evidence of nephrocalcinosis ^(25)^. The efficacy and dosage had not settled in adults with HPP. The efficacy of asfotase alfa for kidney stones and renal failure has to be investigated in the future.

## Article Information

### Conflicts of Interest

None

### Acknowledgement

We thank Helena Akiko Popiel, PhD of Center for International Education and Research, Tokyo Medical University for English-language review.

### Author Contributions

HK, AS, and YO are the physicians and TN is the orthopedic surgeon who handled the case. HK conducted molecular genetic analysis. AS and YO provided critical comments. HK and AS wrote the manuscript. All authors read and approved the final manuscript.

### Approval by Institutional Review Board (IRB)

This study was conducted according to the guidelines of the Declaration of Helsinki and was approved by the Ethics Committee of Kohsei Chuo General Hospital (study approval no.: 2023-9).

### Informed Consent

Written informed consent for publication of this case report and any accompanying images was obtained from the patient. A copy of the written consent is available for review by the Editor-in-Chief of this journal.
